# Who pays for carbon dioxide removal? Public perceptions of risk and fairness of enhanced rock weathering in the UK

**DOI:** 10.1057/s41599-025-05384-9

**Published:** 2025-07-04

**Authors:** Kate O’Sullivan, Nick Pidgeon, Karen Henwood, Fiona Shirani, Harriet Smith

**Affiliations:** 1 School of Geography and Planning, Cardiff University, Cardiff, UK; 2 School of Psychology, Cardiff University, Cardiff, UK; 3 School of Social Sciences, Cardiff University, Cardiff, UK

## Abstract

Carbon dioxide removal (CDR) is increasingly recognised as necessary to achieve net zero emissions. Enhanced rock weathering (ERW) is a novel CDR approach with large mitigation potential. With undefined social and environmental costs, and evolving economic and governance systems, ERW raises issues for public acceptance. A literature gap exists regarding how local communities view the possibility of at-scale deployment in their area. We address this with data from five UK public workshops to identify conditions under which communities potentially impacted by ERW consider deployment fair and acceptable. We show that public acceptance is conditional upon place-sensitive deployment pathways that reflect how place is valued. Analysis highlights opportunities to minimise risk and maximise benefits experienced locally; the importance of transparent governance and monitoring; and unbiased, balanced communication of impacts. Conversely, our research also reveals conditions that might make deployment unacceptable to local communities, including being ineffective as a CDR, environmental contamination connected to ecosystems, the absence of remediation plans, and mitigation deterrence through false carbon accounting. Understanding and incorporating public perceptions and preferences into emergent governance systems is essential if they are to be fair, effective and representative of societal concerns.

## Introduction

Reducing atmospheric carbon dioxide (CO_2_) is increasingly seen as essential for mitigating climate change (IPCC, 2023). To achieve net zero CO_2_ emissions by 2050 the pace of reductions must accelerate (CCC 2024). Carbon dioxide removal (CDR) refers to anthropogenic activities removing carbon dioxide (CO_2_) from the atmosphere and durably storing it in geological, terrestrial, or ocean reservoirs, or in products (IPCC 2021), which is necessary to meet climate goals (CCC, 2025). CDR can fulfil three major functions: reducing near term net emissions, counterbalancing hard to treat residual emissions to achieve net-zero CO_2_ emissions in the medium term, and delivering net-negative emissions in the longer term (Smith et al. 2024:19). Public acceptance is a critical issue for all emerging CDR approaches (IPCC 2023), but with very few exceptions, e.g. (Thomas et al. 2018), existing studies of CDR perceptions typically involve representative national surveys (Wright et al. 2014; Pidgeon and Spence 2017; Wolske et al. 2019; Low et al. 2024), or samples of citizens in workshops and focus groups (Cox et al. 2022; Fritz et al. 2024) evaluating generic risks, benefits and consequences of the approaches. There is a gap in the literature regarding how place-based communities view at-scale CDR deployment, including perceptions of barriers and opportunities, informed in part by experiential and emplaced ways of knowing. We address this research gap through a series of public workshops exploring public conditions for potential local deployment of enhanced rock weathering (ERW), a novel CDR, at an early stage of its development.

ERW removes CO_2_ from the atmosphere by increasing the speed and scale of rock weathering processes that occur when basalt rock is exposed to rainwater (Beerling et al. 2018). Through grinding basalt to granular size then spreading onto land, the contact area between rock and rainfall is increased, as is the rate of weathering and CDR (Beerling et al. 2024). Once weathered, CO_2_ as a bicarbonate solution is transported through waterways to the ocean where it is stored permanently (Eufrasio et al. 2022), potentially also reducing some of the ocean acidification impacts of climate change (Beerling et al. 2024). Modelling for the UK indicates that ERW could remove between 6 MtCO2e and 30 MtCO2e per annum by 2050 (Kantzas et al. 2022). As a result, the UK Climate Committee’s Seventh Carbon Budget makes the conservative assumption that ERW (together with biochar, another land-based CDR) will contribute up to 3% of UK CDR by 2040 rising to 8% by 2050 (CCC 2025). However, for ERW to be included in the UK GHG inventory it is recognised that there is “considerable policy and monitoring, reporting and verification (MRV) development needed” (CCC 2025:273). Given this anticipated contribution and the need to develop long-term deployment governance, it is timely to consider the potential impacts of ERW deployment.

The agricultural and mineral extraction sectors are seen as playing a key role in ERW deployment. Existing agricultural land and transport infrastructure, mineral industry supply chains, equipment and skills within agriculture align well with those required for ERW (Beerling et al. 2018). Other elements in basalt can benefit soil health and crop yield, potentially reducing application of fertilisers or soil conditioners (Beerling et al. 2024). Indeed, research demonstrates that co-benefits for soil and crops associated with land-based CDR – in this case, soil carbon sequestration – are perceived more favourably by farmers than the economic incentives (Buck and Palumbo-Compton 2022). With ongoing field-trial based research regarding the efficacy of ERW as a CDR and its co-benefits for soils and crops, there remain questions around possible environmental and societal impacts of ERW if applied at scale and in the longer term. ERW is often framed as a potential means for farmers to increase their crop growing efficiency and diversify their incomes through carbon trading, whereby polluting businesses offset their carbon emissions by purchasing carbon credits or carbon units (CUs) from net negative emission CDR projects (Joppa et al. 2021). In this instance, farmers could either offset their own emissions or sell CUs to other polluting businesses. However, non-standardised accounting, certifying methodologies, and regulatory gaps in the carbon market mean the price of CUs and purchase conditions are variable (Joppa et al. 2021; Hickey et al. 2023; Santos et al. 2023). Both CDR and carbon trading more generally have been subject to criticism concerning the trade of poor-quality CUs and greenwashing (Low et al. 2024), or because offsetting might operate as a form of ‘mitigation deterrence’ (McLaren 2020; Price et al. 2024) whereby polluting activity continues as usual without efforts to fully address CO_2_ emissions at source (Cox et al. 2018). As such, at-scale deployment of ERW on UK agricultural land holds environmental, financial and reputational risks.

ERW deployment will involve and impact “a diversity of constituencies” (Morris et al. 2024:1169), which, in addition to farmers and land-managers, includes members of the public, and local communities. As a land-based CDR, ERW will be deployed in and adjacent to ‘places’; locations that hold value and meaning to people (Parkhill et al. 2010; Adger et al. 2017; Nicolosi and Corbett 2018). For communities located in places implicated in the ERW lifecycle, social and environmental impacts might necessitate difficult place-specific trade-offs that raise ethical, legal and moral implications (Lawford-Smith and Currie 2017; Cox et al. 2018; Low et al. 2024; Kaltenborn et al. 2020). Research has demonstrated that situated cultural knowledge and views around climate change inform how new CDR technologies are perceived by the public (Minx et al. 2018; Spence et al. 2021; Buck 2022). Furthermore, moral and ethical deliberations around possible place impacts are “situated in specific social contexts” within place (Barnett et al. 2016:2). Through engagement with communities that foregrounds place-based experiential knowledge, it is possible to understand the ways in which communities themselves know and value place (Parkhill et al. 2010; Buck 2022; Fritz et al. 2024). This in turn can elucidate how climate interventions either challenge or support what is important, valued and characteristic about the places people live.

International research involving focus groups in over twenty countries highlights how participants question possible “spatial trade-offs” between farming, ecosystem services, recreation and ERW (Low et al. 2024:6). In such instances, participants suggested that consultation and compensation was needed for affected communities and places. Considering how places may be impacted and what trade-offs might be made requires an understanding of what will be implicated, and how it is valued by communities (Kaltenborn et al. 2020). Thus, while ERW might hold societal benefit, through addressing climate change, it might hold localised impacts (Fritz et al. 2024) or “burdens” (Garvey et al. 2022:1) experienced or paid for, by host communities (Adger et al. 2017). Participants in Fritz et al. 2024 research exploring CDR and solar geoengineering highlighted how different technologies might impact upon local places and communities; and in particular, hold negative impacts on local populations’ cultures and ways of living. As Adger argues, “when the burden of adaption is perceived to be unfair, then action will not be legitimised and interventions simply will not happen” (2016:A3). Accordingly, it is important that insight is gained into how different aspects of place are valued to understand how impacts might be perceived as fair costs or benefits, or where action would not be considered legitimate.

In international research with community members about ERW deployment in Malaysia, participants reflected on their knowledge of local mining activity and experiences of environmental impacts to weigh up the overarching purported societal benefits of ERW with possible adverse environmental consequences that would be experienced locally (Cox et al. 2025a). Cox et al. (2022) also highlight how local natural environments alongside local business viability are valued by communities and are important considerations for evaluating the potential costs and benefits of ERW. Upstream engagement with local communities – defined as dialogue and debate with citizens well in advance of final technology development or any decisions about deployment (Rogers-Hayden and Pidgeon 2007) – can reveal where a technology might present opportunities for remediating locally held place-specific concerns, possibly altering the balance between perceived costs and benefits. For example, in her research exploring community perspectives of a proposed CO_2_ pipeline in Texas, USA, Buck (2022) reports that possible employment opportunities from constructing and maintaining the pipeline was perceived by some as a means of counteracting rural depopulation, contributing to the longer-term sustainability of the host communities. However, Buck’s participants also questioned the underlying governance and rationale for such a project, raising ethical and moral concerns around the distribution of local environmental cost (through digging trenches for the pipeline) compared to the economic benefit that might be experienced elsewhere by corporate owners of the project. This range of research demonstrates how communities draw on experiential and emplaced knowledge and value to consider how different climate change interventions might impact the place they live. Perceived impacts can be made up of both positive and negative outcomes that the community themselves are best placed to weigh up. Such inquiries do more than place people-centredness at the heart of problems-focused risk research. They can elucidate how to navigate questions of intelligibility which arise for researchers as they seek to account for risks’ shifting spatialised and temporalized meanings (Henwood 2022). Such meanings carry affective as well as moral and political loading, which potentially generate contestations over diverse and situated “logics of precariousness” (Switek et al. 2022).

Overall, the exploration of potential climate interventions with in-situ local communities can challenge the “expertise of out-siders” (Buck 2022:1097) and normative framings of “win-wi” (Apostolopoulou 2020:346), through identifying wider ethical, sustainability and practical issues that might affect the efficacy and/or legitimacy of a project. In particular, it can reveal perceptions of asymmetry between the distribution of economic, social, and environmental risks, costs and benefits and how this might lead to rejection of a project by affected communities (Zandlova and Cada 2023) or the development of projects that are “inappropriate for local realities” (Adger et al. 2021:683). Moreover, it can address this through identifying with communities how governance systems and decision-making can incorporate different forms of value and realign decision making to be fair and acceptable (Bennett and Satterfield 2018; Buck 2022; Fritz et al. 2024). As social acceptance of climate change interventions is essential for their successful deployment (IPCC 2023), it is important that deployment plans incorporate where possible economic, social and environmental conditions derived from affected citizens. Below we set out how we sought to address these issues through our detailed place-sensitive workshops.

## Methods

Our research explores public conditions on potential local deployment of ERW in the UK as part of an interdisciplinary research consortium programme relating to ERW. Workshops explored how participants understood ERW when placed into a local context, and investigated possible opportunities, risks and benefits for the places that people live in now and in future. In this paper, we seek to address a central research question; how can upstream social intelligence elucidate deployment barriers and opportunities for ERW situated in local places?

### Case selection and recruitment

**Fig. 1:**
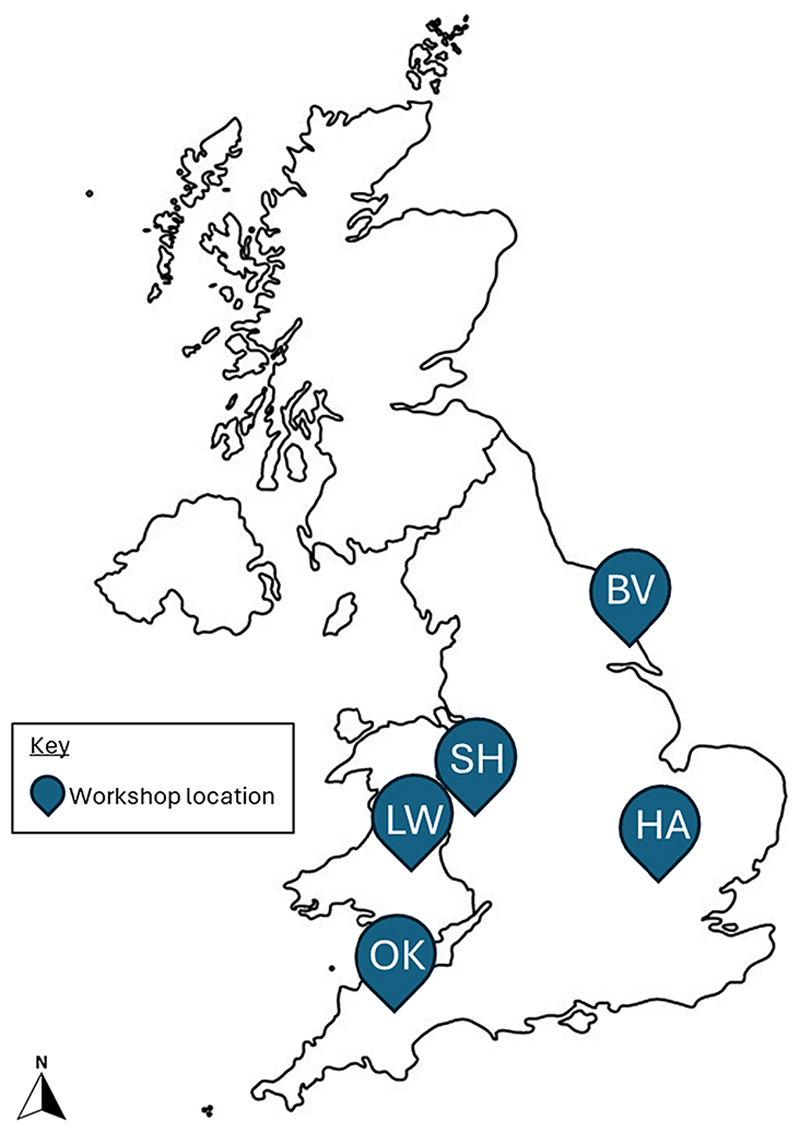
Locations of the five UK workshops.

Workshops were conducted in April and May 2024 in five locations across England and Wales, purposively selected due to their different agricultural land types and relationships to the ERW lifecycle (Table 1, Fig. 1). Workshop location selection was informed by existing literature identifying potential deployment locations (Kantzas et al. 2022; Madankan and Renforth 2023), spatial data from the Office for National Statistics and DigiMaps, and geodemographic data from Consumer Data Research Centre, as well as through discussion with the wider Enhanced Rock Weathering - Greenhouse Gas Removal Demonstrator (ERW-GGRD) consortium.

The workshops sought to explore potential impacts of ERW in place, drawing on communities’ shared cultural knowledge, traditions, history and lived experiences (Buck 2022; Thomas et al. 2020). Therefore, participants were recruited from within a ten-mile radius of the workshop location, ensuring they held some locational familiarity that could enable place-specific deliberations. Llandrindod Wells (LW) and Okehampton (OK) were selected as population centres proximate to ERW demonstrator sites. Both locations represent rural towns and sparsely populated fringe areas in agricultural communities with a nearby basalt quarry (within 15 miles). Harpenden (HA) was also selected due to proximity to a demonstrator site, but represents a peri-urban area, within commuting distance to London. The three demonstrator sites differ in their agricultural use and land type, with ERW being tested on upland livestock grazing in LW, lowland pasture in OK, and arable cropland in HA. The Vale of York is identified in ERW deployment models as a location of medium-term and continued deployment of ERW. Within this county, Beverley (BV) represents a mix of rural and urban populations, has a high level of arable farming and is near to the coast and Hull dockland, implicated later in the ERW lifecycle. Finally, Shrewsbury (SH) was selected as a mixed farming area (both arable and pastural), close to two basalt quarries, and identified as a location for potential early ERW deployment. All five workshop locations are near to or have rivers running through them, which, given the potential impact of ERW on watercourses, was an important criterion for location selection.

Within the specified location, each group was recruited to ensure even sex representation, as well as a diversity of age and socio-economic status. Ethnic diversity reflected the local population for each group, with 44 of the 48 participants describing themselves as White British. Workshop groups involved 9–10 participants; 10 were recruited for each group but two did not attend. Participants were recruited via a professional recruitment agency using a combination of face-to-face and online methods. Recruitment techniques varied according to the area characteristics but included recruiters being present in local towns and population centres. Recruiters used a screening questionnaire and participants self-reported demographic information. Potential participants were informed that they did not need to know anything about ERW to take part. Each group involved a diverse spread of community members living in each place, selected to ensure a broader range of issues in relation to ERW would be discussed. A small number of participants in the study (n = 8) had experience of or direct connection to the agricultural sector (as farmers or within the supply chain). During some points in some workshops, particularly in relation to more technical information, participants with agricultural connections contributed more readily to discussion. However, as per Macnaghten (2020), the experienced facilitation team made efforts to ensure that all participants had full opportunity to share their thoughts. Participants received £125 for taking part.

### Data collection

**Fig. 2:**
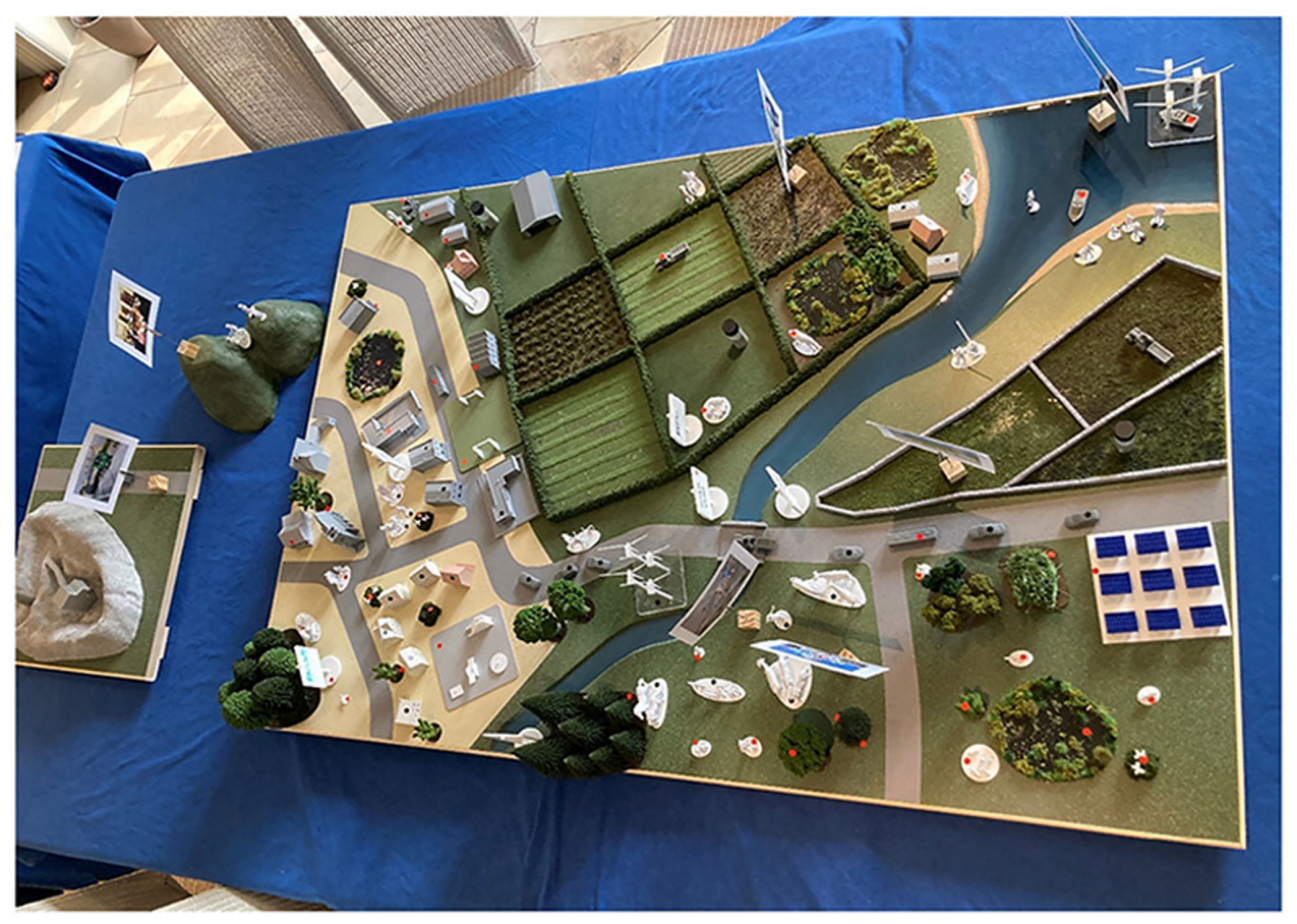
Populated model (BV).

The workshops incorporated a range of activities, involving information provision and interactive tasks, with opportunities for group discussion. Activities were designed to provide insight into ERW – which, similar to other CDR strategies, was something the majority of participants were unfamiliar with (Pidgeon and Spence 2017; Spence et al. 2021) – and provided opportunities for participants to think about different phases of the ERW lifecycle. A focal aim of the workshops was to encourage reflection on potential impacts of ERW within the context of the places in which participants lived. To facilitate this, we utilised a bespoke 3D landscape model designed to include key landscape features important to the ERW lifecycle and which could also be related to recognisable elements of each workshop location. This included generic features such as a river leading to coastline; arable land; pastureland; urban area and road demarcations. Participants customized the model to represent the place where they lived and what was valued by them using moveable components, ranging from large elements such as hills and a quarry, to smaller pieces representing buildings, infrastructure, vehicles, people, animals and vegetation (Fig. 2). While components were clearly labelled, participants were able to interpret the pieces differently and were also given blank ‘sign-post’ pieces they could label to reflect anything they felt was important locally but not represented by the pieces supplied. The landscape model provided a means of emplacing participants’ thoughts and acted as a setting for understanding possible land-scape change and other impacts that may result from ERW deployment.

The first workshop session (S1) focused on exploring participants’ emotional and experiential understanding of place. This session aimed to both act as a form of icebreaking enabling participants to gain confidence in communicating with each other and researchers, and to develop a shared understanding of the place they lived in. Additionally, this initial exploration of place provided a setting for later discussions about possible local impacts of ERW. To enable this, we used the landscape model to consider physical, social and cultural landscapes. Participants were asked to reflect on landscape features that they considered important to, and were recognised more broadly across, the community. In doing so, participants talked through their own personal perspectives, feelings and experiences, while also engaging meaningfully with community landscape perceptions in terms of their local and wider relevance. During initial discussions, a member of the research team populated the model with components to reflect what was being raised, positioning pieces on the model in agreement with participants. Following this, participants were invited to select their own components to further populate the model, with each person explaining in turn the reasons for their selection, which could represent something they perceived as important to the community or to them personally.

The second session (S2) focused on the presentation of information about ERW, describing what the process involved, the current state of knowledge and possible implications, including where questions were still being researched and outcomes uncertain or unknown. Information provided was neutral and balanced, avoiding known frames such as the nature-technology divide in valuing CDR (Cox et al. 2025b) and overestimations of potential emissions-reduction (Bellamy and Raimi 2023) that could shape the outcomes of discussions (Braun et al. 2018; Wolske et al. 2019; Spence et al. 2021). For example, we were careful not to frame ERW as a ‘natural’ weathering process, although this term was used by participants in their discussions. When explaining possible CDR or other co-benefits (to soil health for example) we noted that data sets were based on small-scale demonstrator outcomes. To convey information about ERW, a combination of video resources, images, and physical samples of basalt were utilised. Participants had the opportunity to ask questions verbally and to note down questions on post-it notes, which were collated at the end of the session. During S2, the research team informed participants that there was different information about costs to farmers and that costs were likely to vary based on factors such as farm location and the price agreed with contractors for basalt and spreading. We also made clear that research is ongoing into the availability of basalt, suitable quarry locations and how viable it may be when working out the cost and CO_2_ involved in quarrying, grinding the stone into small pieces, and transporting it to farms. Overall, we conveyed that this meant potential costs were uncertain.

**Fig. 3:**
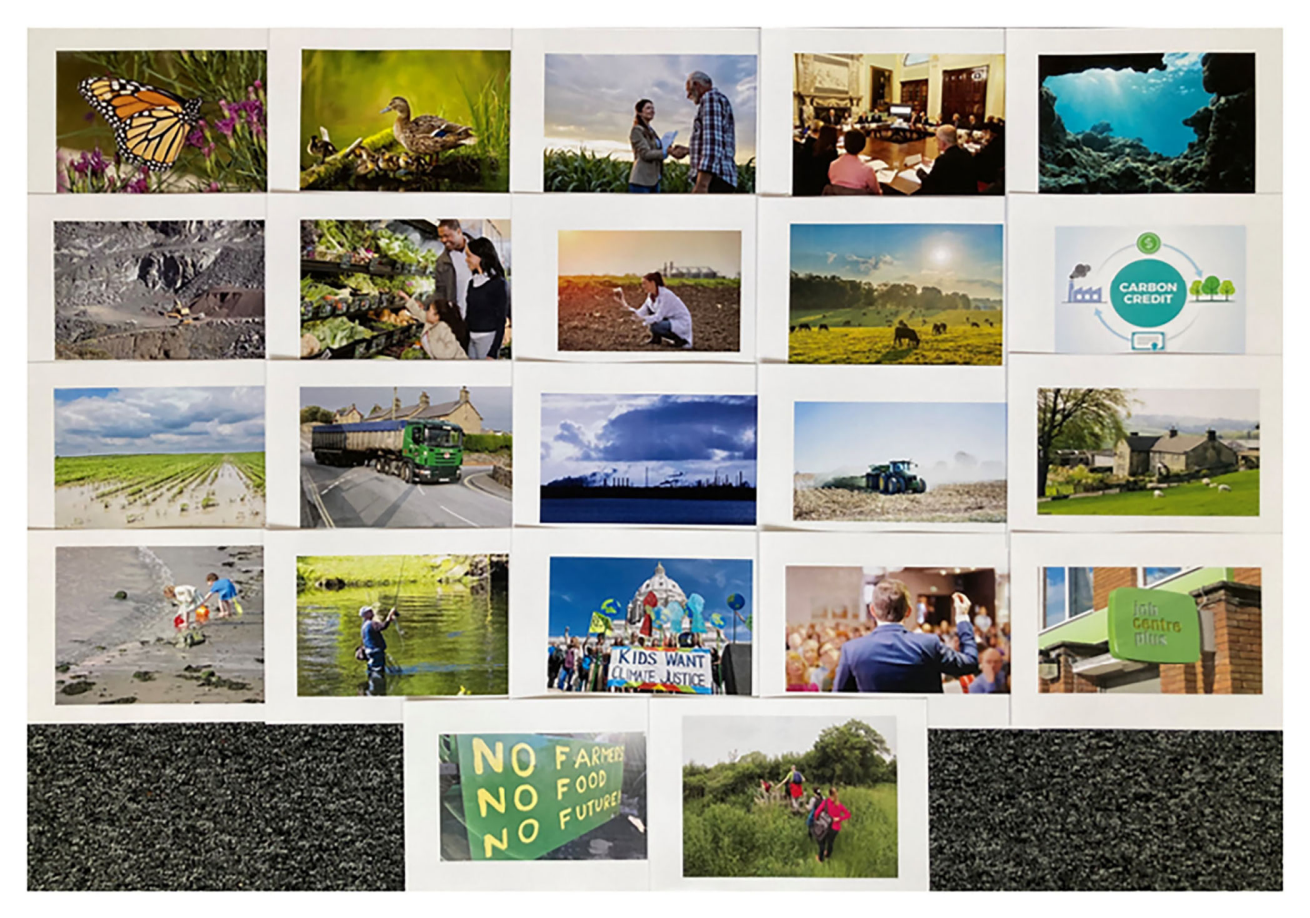
Selection of images – angler fourth row, second image from left; quarry second row, first image from left.

**Fig. 4:**
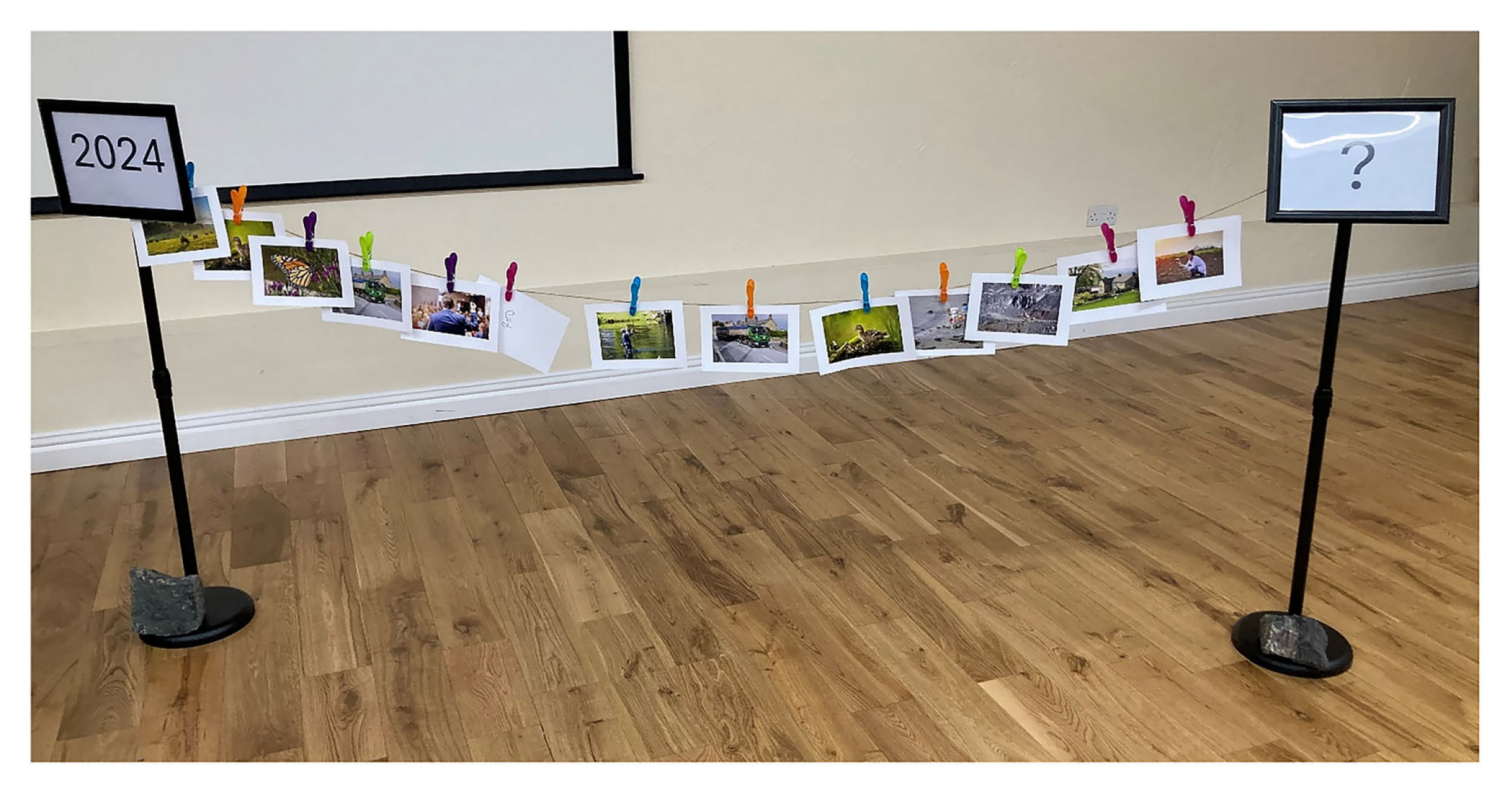
Completed string line (OK) – Harrison’s drawn image sixth from left.

Following this (S3), participants were given the opportunity to ask further questions before the researchers gave a brief overview of actors who may be implicated in the development and deployment of ERW in the UK, which included information about carbon trading. The second part of S3 involved an image-based activity. Each participant was asked to select an image that represented something they felt was important in relation to ERW (Fig. 3). If participants felt that none of the available images represented an issue they would like to raise, they had an opportunity to write or draw something on a blank piece of card. Each participant in turn was asked why they had chosen the image and what they felt it represented. Participants were then asked whether they felt the issue in question was an immediate priority or a longer-term concern. Based on this temporal reflection, the participants, collaboratively with the research team, decided where to place the image on a stringline, representing the potential trajectory of ERW into an unknown future period (Fig. 4). Once each participant had selected and discussed an image, they had the opportunity to select further images for inclusion, until a comprehensive range of issues had been represented.

The final session of the workshop (S4) returned to use of the 3D landscape model, to explore how participants imagined ERW deployment might impact place, including ethical concerns around local trade-offs, aesthetic and symbolic values, and cost-benefit analysis. For this final session, the research team added vials of basalt in various places on the model to represent where basalt may be quarried, transported and spread. If not utilised in early discussions, the model quarry was also placed on the table to represent the role of quarrying in ERW. Miniature versions of the images from S3 were also available to participants to represent important issues and were placed on the model during relevant discussions. This workshop session drew on earlier discussions, bringing together issues raised to explore the potential repercussions of ERW in local place context.

### Analysis

Workshop discussions and activities were audio and video recorded, with additional researcher notes and photographs taken throughout the workshops of the model and image line. Following each workshop, photographs were uploaded and labelled, audio files were transcribed by a professional transcription company and then checked for accuracy by the research team using the video recordings. During this initial check, non-verbalised detail was added to the transcripts (e.g. nodding, pointing to particular aspects of the model, identifying images selected by participants) given this provides important context and can fundamentally alter interpretations (Matoesian and Coldren 2002). All transcripts were anonymised, and pseudonyms are used for participants throughout this paper, alongside workshop session identifiers. Where discussion arose in relation to the image activity in S3 we reference this, otherwise the data presented arose from more general discussions and question and answer opportunities.

Initial analytic steps involved individual team members making research notes and subsequently sharing interpretations and perceptions of connections between emerging insights as a research team. This allowed us as individual researchers to become familiar and immersed in the data (Turner 1981; Braun and Clarke 2022), while also bridging gaps in our interpretations or making connections between different researchers’ insights. We adopted a thematic analysis approach as a form of data categorisation to capture important themes within the data (Maxwell and Chmiel 2014), using inductive codes to map out the main themes arising from workshop discussions. Coding followed an iterative process involving multiple readings and interpretation of the dataset (Charmaz and Henwood 2017). Themes were developed by considering patterns in the coded data that reflected substantive ideas (Glaser and Strauss 1967) or a shared meaning (Braun and Clarke 2022) and not by using a pre-determined framework (Henwood and Pidgeon 2003). These themes were reviewed to ensure the coded data was reflective of the idea they represented and to enhance our understanding and interpretation (Braun and Clarke 2022). In this paper, we draw on data that comprises three themes relevant across the whole dataset, derived by inductive qualitative analysis within and across workshop data. Firstly: ‘governance systems - who pays?’ comprising participant discussions of how ERW deployment might be financed and associated ethical considerations around where or with whom responsibility for CDR sits, what is valued and by who, and possible consequences associated with different financing routes. Secondly, ‘cost and benefit of ERW’ comprising discussions around perceived economic, environmental and social impacts for place including deliberation of possible trade-offs and/or opportunity to enhance benefits. Finally, ‘risk and temporality’ a theme reflecting discussions of evidence of ERW efficacy, displacement of impacts of deployment (or non-deployment) to future generations, monitoring, verifying and reporting and later remediation. We recognise that our findings are influenced by our research design and method choices, by how participants engaged in the research process and what they chose to share with us at that time, the wider context in which the research has taken place, and through our interpretation of the data which will always be informed in some ways by our own subjectivity (Braun and Clarke 2022). We have prioritised communicating accessibly and meaningfully how we have made conscientious efforts to provide an authentic, truthful way of reading our data, rather than demonstrate new forms of reflexive practice informed in some ways by our own subjectivity (Gergen and Gergen 2000).

## Results

In this section, we present data relevant to our research aim to elucidate deployment barriers to and opportunities for ERW through upstream social engagement. Our emergent analytic approach enabled us to identify how participants used both general, high level societal, political and economic perspectives alongside their emplaced experiences and knowledge to understand ERW and discuss potential impacts. Discussions in all the workshops encompassed ethical questions of fairness and distribution of cost and benefits. At a societal, political and national economic scale such discussions involved consideration of fair financing for ERW. In these discussions, perception of risk and benefit for local farms was a salient issue, alongside societal goods and concerns for transparent and legitimate corporate finance routes. Characteristic and intrinsic qualities of place, such as local natural environments and wildlife or the sense of place evoked through perceptions of air quality for example, were drawn on across all the workshops to illustrate concerns for local social and environmental impacts. These exemplify both impacts that might be experienced within place, for example, places in close proximity to quarries would likely be most impacted by increased quarrying and transportation of basalt, but also at a societal scale, for example, the loss of experiences in nature for future generations through unintended harm to biodiversity. The likelihood of ERW impacting the locations where participants lived varied according to their proximity to proposed ERW deployment locations and how ERW was perceived to support or pose risk to currently valued aspects of place. We begin by highlighting common concerns raised across all groups before moving on to explore place-specific issues.

### Financing ERW: risks, responsibility and the ‘greater good’

Common across all the workshops were pragmatic and ethical discussions around how ERW deployment in the UK might be financed and who should be responsible for this. Three main financing routes were discussed: independent investment and deployment by farmers, public funding via subsidy, private investment via carbon trading. In discussions participants weighed up the distribution of risk, fairness and concerns for unethical practice. In all workshops, participants acknowledged the role that agriculture plays in both providing food at a societal scale, and managing landscapes and contributing to communities at a local scale. Some participants referenced how historical farming practices had been ecologically extractive and how increased emphasis on regenerative farming – farming practices that work in synergy with nature and natural processes – was welcome. In this framing, possible co-benefits for soil health through a ‘natural’ (Harrison, OK) weathering process was perceived positively. However, participants questioned whether it was fair for farmers to pay for and deploy ERW on their land given limited research evidence of long-term ecological impacts. Accordingly, participants raised concerns about the potential risk to farmers’ livelihoods and reputations should things go wrong:

If I was a farmer, I’d want some sort of assurance that it weren’t gonna damage my land if I’m doing this for the greater good, it’s not gonna damage my land, which is my livelihood. (Rhys, LW, S3)

ERW was perceived as an immediate additional financial cost with uncertain risks and benefits for farms. One participant described this as akin to asking farmers to accept a ‘pay cut’ (Daniel, SH). Some questioned whether ERW would take land out of productive agricultural use for any period of time, which could then have economic consequences for farmers:

(w)ill they be able to use the fields for anything else while that’s sitting on the top now, or that will actually write off that field for the length of time until the rain got on it? … And they wouldn’t make any money off it while it’s resting or whatever. (Liz, OK, S3)

As ERW was perceived to be addressing a societal scale issue it was seen by some participants as unfair for farmers to take on the risk of paying for deployment given it was for ‘the greater good.’ Thus, a second route involved society paying for ERW deployment, either through cost ‘pass-through’ (via increased consumer food and water prices) or publicly funded government subsidies. Participants discussed how agriculture was heavily subsidised, yet many farms struggled to remain viable. While some expressed reluctance to increase public funding, this was set against reasoning that without the financial incentive that subsidies offer, farmers would not consider ERW at all:

Most people are working to make a living. Farmers are no different to anyone else. So it’s got to be profitable for them. And it’s only going to be if they’re getting money from the government. (Allan, HA, S3)

Increased costs to the public were perceived as inevitable if farmers or water companies incurred additional operational costs. In the image task, only one participant across all of the workshops explicitly raised the issue of cost - using a blank card to draw a pound sign and explaining his reasoning for this:

Partly from the point of view of the farmers and myself etcetera, to be quite interested in if it’s good, cost, and I also wrote down actually thinking of South West Water, are they gonna have additional processing costs that should have been passed on to the consumer for filtering it out? … that is gonna impact everybody in here if the cost of water went up because South West Water suddenly gained an additional cost, they always belong to the consumer at the end of the day, fairly soon (laughter). Yes, it’s one of the things, isn’t it, to think about if they, they’ve got to filter it out. How much are these filters gonna cost in the grand scheme of things, and so much to increase the costs for all the general public paying for water every month. (Harrison, OK, S3. Fig. 4)

Harrison’s quote suggests not only anticipated public costs, but differences in how externalities such as downstream pollution costs are borne geographically; with consumers in areas proximate to ERW deployment potentially seeing higher bills as a consequence.

The final route discussed was ERW being funded by private corporate investments via the purchase of CUs to offset business CO_2_ emissions. In all workshops, participants’ perspectives aligned with the “polluter pays principle” where the responsibility for the costs of pollution should be borne by those who have caused it, and not those impacted by it (DEFRA 2023). Offsetting emissions through investing in ERW CUs could support this. While most participants were unfamiliar with the idea of CUs or offsetting, which were briefly explained by the research team, some described examples of poor-quality CUs. This contributed to expressions of mistrust in carbon trading and raised questions around governance:

There’s a lot of issues in the media about it. Where like your big corps are buying up these carbon credits. And they’re like reforestation projects. But then in five years’ time, they cut down the trees, and then they just have more land, so they make new credits up. So, it’s like, who’s actually controlling that? (Robin, HA, S3)

For some, CUs could provide a means for polluting companies to continue to work in the same way, and offsetting was perceived as defeating the purpose of deploying ERW or being a “lazy option” (Sam, HA, S3). However, most participants were comfortable with carbon trading if the corporate CU buyers were already reducing their emissions and this was “an additional thing” (Anita, BV, S4). Participants perceived a possible financial benefit for farmers if they were able to sell CUs generated from ERW and so this was seen as a fair way of paying for ERW.

Overall, while there was concern that offsetting CO_2_ emissions through purchasing ERW CUs might be a form of mitigation deterrence through direct carbon substitution, most participants across the five workshops also expressed the view that those who produce CO_2_ emissions should be responsible for paying for CO_2_ removal or mitigation.

### Emplaced costs: environment, disruption and remediation

Underpinning discussions of responsibility, risk and fairness for different ERW finance routes were questions and concerns regarding environmental and social risks. Participants expressed unease at limited research data evidencing environmental impacts that may adversely affect soil and water ecosystems and biodiversity longer-term. Some, like Rhys below, drew on their emplaced knowledge and experiences of nature and wildlife to highlight how this was valued, as intrinsic aspects of the place they lived, and in this instance part of the community’s cultural heritage:

We’ve got so much flora and fauna and insects around us that we don’t want to get spoiled or lose. And again, like if you were to roll this out into Elan there’s parts of Elan Valley (in mid-Wales) that are scientific interest areas (…) they’ve got their natural rainforest and all that there. That’s the stuff we don’t want to lose because that’s our heritage, we’ll never get it back. (Rhys, LW, S3)

While specific examples varied between workshops, depending on the landscapes, habitats and species native to each place, a commonality across all workshops was the high value participants placed on natural features and wildlife. For many, nature and wildlife was discussed as providing variety, colour and enrichment to their lives, and so it was perceived as important to preserve wildlife so that future generations could form similar relationships with nature. These discussions were particularly prominent in session 3 in response to the nature-based images. Unknown impacts associated with ERW were perceived as a risk of potential environmental damage, which would impact participants and future communities. Selecting the image of an angler (Fig. 3), Mac described his choice of image in relation to questions about how possible environmental costs of ERW were considered in the form of trade-offs:

Does (ERW) have an impact on the water quality in the rivers and will that affect the biodiversity in that river? (…) it’s not just the fish life, it’s the plant life, it’s insects, it’s, it’s the amphibians that live in these environments, it’s the mammals that live in these environments that it could be affecting and impacting (…) does that offset the benefits of sticking the basalt on the ground? (Mac, SH, S3)

Others reflected on environmental disruption from quarrying activity, something likely to expand with at-scale deployment of ERW (Kantzas et al. 2022; Madankan and Renforth 2023). Participant concerns encompassed both material impacts of extraction on the landscape, and ethical concerns for spatial and distributive justice where communities proximate to quarries would bear particular costs and environmental impacts:

One of our concerns was who pays? You know, who pays, who benefits? Is it going to be a national scheme or is it going to be left to industry where shareholders are going to benefit, and we’re going to have the costs of living with gravel pits and quarries all around us, and heavy traffic on the roads. (Teresa, BV, S3)

While quarrying expansion was generally viewed as likely to have a negative impact for local communities, from pollution and transport disruption to potential impacts on house prices, there were some positive impacts associated with possible local employment opportunities. Place-specific contexts such as the presence of quarries and condition and character of local transport infrastructure affected participants’ perceptions. Participants in LW expressed concern for basalt transportation by drawing on present experiences regarding road damage, light pollution and safety concerns for people and animals due to large logging trucks currently driving through small villages on narrow winding roads. In HA, several participants spoke of their decision to live there informed by intrinsic and environmental qualities such as “cleaner air, greenery” (Julie, HA, S1). Here, basalt transportation using haulage trucks was perceived as directly impacting these through increasing “emissions and pollution” (Julie, HA, S3). In contrast, the SH group reflected on current experiences of local quarrying activity in Shropshire where the quarry was perceived as being well managed in terms of minimising community disruption from blasting noise or transportation. Similar to findings of (Parkhill et al. 2010:53) perceptions of local impacts due to increased quarrying were made “reflexively”, informed in part by familiarisation with the local quarry and mineral transportation. Thus, several participants here perceived that quarry expansion might not adversely impact the area:

Olivia: We’re fortunate enough to have some quarries within Shropshire. That’s one thing in terms of transportation, that’s locally. We wouldn’t have to get it transported in as such, maybe.

Simon: I don’t think we’d notice much difference about the traffic either, to be fair. I don’t think it would impact too much.

Dominic: And then, like the trucks, they’re not allowed to go through the main town or anything anyway, are they?

Daniel: No, that’s right, there’s weight restrictions on most of the roads. (SH, S4)

Concerns about environmental disruption and geographical inequities were raised in all workshops in reference to ERW deployment but also “post-implementation” (Lawford-Smith and Currie 2017:2) in terms of decommissioning and land remediation for quarries, and farmland should ERW cause damage in the longer term. After initially choosing an image to represent information and decision-making, Jacob chose the quarry as a second image to highlight longer-term concerns regarding remediation:

What’s gonna happen to quarries, you know, that’s a main concern when it’s all done, when it’s all dug up where you leave the quarries? (…) Are we just gonna have loads of holes in our country? (Jacob, OK, S3)

Overall, participants highlighted several possible environmental and social risks associated with ERW, leading them to weigh-up the trade-off between the benefit of CDR and the risks of unknown costs in their locations, both of which would only be fully realised in time.

### Public conditions for effective and fair ERW deployment

Through weighing up possible risks associated with ERW and how these might be distributed between different actors and affected places, conditions for fair ERW deployment emerged from the workshop discussions.

Participants saw multiple opportunities for ERW to hold benefits for place. All groups raised potential job creation as a benefit, while some groups raised specific local issues. For example, BV participants suggested investment in basalt transport infrastructure for deployment across the extensive farmland there could not only revitalise the existing local docks in Hull, but it might also be possible to ensure funding is allocated to address place-specific flooding concerns:

But if you do it in the waterways, you can transport vast amounts on ships, and it will come in that way. And they invest in the waterways, which then probably get more invested in the dredging and the flood defencing, which is probably might counter some of the problems that you’ve got now. (Richard, BV, S4)

Alongside potential large infrastructural benefits of the kind Richard envisaged, some groups suggested that because costs of ERW would be borne by particular communities, they should also get direct community benefits to ‘soften the blow’:

(y)our way around that one is like, you got Builth Quarry there now, if this happens, put a pound a ton on, and give it to the local town again. They want the new rugby pitch, there it is, or 50p a ton, they would be taking tons out, it would mount up, wouldn’t it? That’s your way to ease the locals. … If you’re buying a house in Builth Wells, you know there’s a quarry there already, don’t you? But it’d be, it would soften the blow, if like 50 pence a ton. (Ken, LW, S2)

While participants saw opportunities for ERW to hold localised benefits for place and ways that possible disruption could be mitigated, such ideas were only perceived as permissible if ERW was an effective CDR and possible adverse social and environmental impacts were identified and addressed. To address these concerns, participants across the workshops considered continued research and long-term monitoring essential. Local-scale monitoring – at places affected by deployment – was perceived as safeguarding against assumptions within national-scale scenarios and modelling that cannot account for different local environmental and social contexts, or unforeseen and unintended local consequences. Continuous monitoring could enable further research evidence to be gathered supporting ERW efficacy while also safeguarding against emerging risks, identifying in real time if ERW deployment should cease:

(i)t absolutely has to have continual monitoring. I don’t think you can have your research projects and suddenly say “this is great, there’s no problem with it.” You can’t just chuck it out to the industry to do and then not notice that actually everything seems to be changing. (David, OK, S4)

Some participants perceived regulation as a mechanism to prevent agricultural land loss, either through unintended environmental damage caused by ERW or through the inappropriate purchase of productive agricultural land solely for carbon trading. In the latter case, the purchase and removal of farmed land for rewilding and carbon trading was drawn on to highlight how ERW might have similar local consequences:

a 750-acre estate in Hereford has just been sold to a multibillionaire guy for his daughter because she liked the house. So all the tenants have been kicked off, all the people who are renting it and growing crops have been kicked off. And they’re going to just rewild it, and do nothing with it but sell the credits to the carbon. Well, 750 acres come out, if we keep doing this, we’re going to have no food. It’s crazy. (Hugh, LW, S2)

Participants argued that as well as its positive potential, negative and unknown aspects of ERW must be communicated transparently to the public. Here, some participants drew on examples of other climate interventions that they perceived had been upsold to the public, only to have sustainability claims downplayed later, a phenomenon similar to the ideas of ‘controversy spillover’ (Cuppen et al. 2020) and ‘ripple effect’ (Cox et al. 2022). While existing negative perceptions influenced perceptions of ERW, participants spoke of improving this by ‘continuous’ and ‘transparent’ (Jacob, OK) MRV, incorporating regular unbiased and balanced communication of research findings.

Connected to discussions of transparent communication were those concerning the integrity of decision-makers and those responsible for developing governance systems for financing, deployment and monitoring. During participant deliberations “no easy answer” was found as to who should be responsible for decision-making and monitoring (Lawford-Smith and Currie 2017:2). Several participants expressed mistrust in government decision-makers, alongside perceptions of vested interests by carbon market actors. For example, BV participants questioned the motivations of decision-makers when it came to costs of ERW deployment, given they were seen to be potential beneficiaries and unlikely to be those bearing the cost:

Clive: I don’t want to get too political, but 14 years of whichever colour you get in, these are the people making the decision, and I don’t trust them to make the decision based on global warming, the economy, and whether it’s better for this country. I don’t think that will be the key thing. It will be the cost and whether they’ve got a company involvement …

Richard: So we need an informed decision given to the public.

Clive: We need an informed decision giving to people who have the country’s best interests in heart…

Richard: Not self-serving.

Teresa: It’s the opposite of who pays, who benefits. And it’s not necessarily us. You know, you need to look at who benefits and who is paying because often the people who pay are not the people who benefit.

(BV, S3)

Together Teresa’s quotes and the initial statement from Clive highlight two key concerns. Firstly, that the cost of ERW is not just financial, but will include non-monetary costs (in this example associated with environmental and social harm from quarry expansion and road damage/traffic congestion and exhaust fumes/air pollution). Secondly that decisions made about whether and where to deploy ERW will be taken by those who will not bear those costs, but who might financially benefit. In partial answer to this, participants referred to environmental regulators and their role ensuring adherence to environmental standards as key to monitoring ERW. Others suggested independent and academic research as able to impartially assess ERW, although given this is often funded via government research contracts, there was scepticism that this may not always be unbiased.

## Discussion

In this paper we have shown how upstream public engagement can elucidate deployment barriers and opportunities for ERW. In doing so, we address an existing gap in the literature regarding the ways place-based communities who might be asked to host CDR interventions will view at-scale deployment. Similar to Buck (2022), Fritz et al. (2024) and Cox et al. (2025a), our research illustrates how participants discussed both general issues and place-specific concerns, for their own and other places. Higher order discussions included societal responsibilities for addressing CDR, the role of and trust in Governments and private markets, and the need for transparent and truthful information provision. Place-specific discussions included concerns for social and environmental impacts from quarrying activity and basalt transport, as well as identification of ways of leveraging jobs and investment or addressing pressing climate and infrastructure concerns in their local places. In this way, workshop discussions encompassed ethical concerns for the potential uneven distribution of spatial impacts as well as uneven temporal impacts whereby deployment of ERW now, could impact both future communities in place and future wider society.

Discussions of higher-level issues were often interwoven with local place-based concerns and issues arising from participants’ knowledge and experience. This is visible in participant discussions around possible impacts to natural environments and wildlife that could be experienced at all stages of the ERW life-cycle. During these discussions participants reflected on risk for both natural environments and wildlife they valued in the place they lived, in addition to perceptions of risk of loss for place and society more broadly. Participants across the workshops spoke of the need for monitoring and verification of ERW, and plans for remediation which would be necessary within local sites of deployment. Like in Buck and Palumbo-Compton (2022) participants expressed concern that this localised process might be untransparent and open to bias if taken forward by central actors, such as the Government, who would not necessarily prioritise local place interests. Participants identified several conditions they deemed necessary for deployment, including local-scale, long-term monitoring. This aligns with recommendations by the CCC (2025). For participants, long-term monitoring was regarded as a safeguard against assumptions within national-scale scenarios and modelling that cannot account for different local environmental and social contexts and unforeseen and unintended locally experienced impacts. Unbiased and balanced communication of findings at all stages of research (Rayner et al. 2013; Spence et al. 2021) was also deemed essential for local communities.

Discussions of financing ERW across all workshops encompassed consideration of not only monetary cost, but possible risks associated with different finance routes. Deploying a CDR with a limited scientific evidence base to demonstrate its efficacy and long-term outcomes was perceived as posing significant risk for farmers, as possible subsequent damage to farmed land would impact farm viability. A key concern for participants was that financing routes were considered to be fair, transparent and geared towards the genuine removal of CO_2_ rather than acting merely to deter at-source mitigation. For these reasons, public funding and private financing via carbon trading were discussed as being fairer. In these instances, the polluter (either society or polluting corporations) was perceived as paying for the removal of CO_2_ that they had emitted, instead of the ‘burden’ of ERW being carried solely by famers not responsible for the emissions. This is reflective of literature regarding mitigation deterrence (McLaren 2020; Morris et al. 2024; Price et al. 2024) and the displacement of risk associated with ERW at an international scale (Lawford-Smith and Currie 2017).

Discussing ERW as a potential CDR with members of the public is challenging given low prior awareness of the topic, which necessitated conveying a considerable amount of information in the workshops. The design allowed for regular questions or clarifications, but some participants may have found it challenging to fully comprehend the amount of information provided. In addition, conveying information may have given the impression of the research team as advocating ERW. However, at the end of each workshop when participants were asked for feedback, the majority indicated that they felt the research team had presented information in a fair and balanced manner. The design of the workshops aimed to focus discussion on potential place-specific impacts of ERW, and participants identified issues relevant to their local places. However, providing information about the ERW lifecycle meant that participants also discussed more general issues with different aspects of the process, as well as potential costs and benefits for other places. Whilst the workshop locations were selected to represent different stages of the ERW lifecycle and deployment trajectory, further work in areas closest to the basalt resource and in the most likely areas for initial deployment could supplement our project findings.

## Conclusion

Our research with emplaced communities has explored possible impacts associated with deployment of ERW, a novel CDR. It shows how public acceptance of deployment at scale is conditional (Pidgeon 2020), and elucidates both generic and specific, locally grounded, conditions on which such deployment would be considered acceptable. These conditions centre around ensuring ERW efficacy as a CDR, that financing of ERW reflects polluter pays principles, and for robust, transparent and trustworthy MRV. Our research also illuminates public questions and concerns, indicating circumstances in which deployment would not be desirable for local communities. Upstream public engagement work like ours can therefore make an important contribution to understanding these conditions and identifying potential risks that should be avoided in ERW deployment. Unacceptable conditions, whilst perhaps obviously incorporating the negation of acceptable conditions, also encompassed other issues. This included: environmental contamination, perceived as hard to reverse and holding wider impacts for interconnected ecosystems; the absence of remediation plans should environmental contamination occur or for ‘end of the lifetime’ and ‘post implementation’; and finally, ERW enabling mitigation deterrence or being used for false carbon accounting.

As ERW is a proposed societal-scale intervention for climate change, public funding and private investments were considered fairer than economic investment by individual farms. Even so, we found that conditions were also attached to these funding options. For example, carbon trading was most acceptable with rules to prevent unscrupulous purchase of agricultural land and removal from food production, to protect farmers’ interests and to ensure that investing companies evidenced they were also “addressing the root cause” (Cox et al. 2018:3) of their emissions. Other conditions stemmed from concerns for possible unknown and unintended environmental impacts that at-scale ERW deployment could hold, possibly impacting local ecosystems, biodiversity and affecting connection to nature and place. As such, a key condition to ERW deployment was the development of a robust empirical evidence base for ERW through time and repeated applications. In the reporting of monitoring and research findings, unanswered questions and negative outcomes should be communicated in the same way as positive outcomes. A key further condition to deployment is that during the development of governance systems, the characteristics, resources and perceived problematic aspects of place (i.e. areas of flood risk or limited local employment opportunities) are recognised. This will foreground opportunities to address place-specific issues and concerns for climate change, local economies and nature. Relatedly, our research highlights the need for clear deployment plans that elucidate why certain places are implicated in parts of the ERW process and how, throughout its whole lifecycle, ERW can be both net negative CO_2_ and economically sustainable. Place-specific deployment plans should be transparent and accessible, identifying accountability, responsibilities and timeframes.

This work makes an important contribution to the ongoing debate about the future of CDR, and the forms of public condition that need to be incorporated into decision-making to ensure acceptable deployment pathways, governance systems and monitoring arrangements (Spence et al. 2021, Fritz et al. 2024). Through foregrounding place-based experiential knowledge, and enabling associated ethical discussions to arise, the study provides insight into how different layers of perceived risks and benefits are understood. Responding to such local knowledge and cultural perspectives can also support the development of appropriate governance processes (Bennett and Satterfield 2018; Buck 2022), a prerequisite for successful CDR schemes. The study reveals how perceived social and environmental risks may be mitigated, and benefits maximised locally, thereby suggesting conditions for deployment that are considered both fair and acceptable by affected communities.

CDR is now a credible policy option for the UK (DESNZ 2023), with projects expected to increase over coming years, while private investment via carbon trading in CDR is already enabling commercial deployment of ERW in the UK. This research provides timely insights relevant to the research community, industry and policy makers responsible for shaping deployment and development of associated governance systems.

## Figures and Tables

**Table 1:** Participant groups.

Group location	Dominant agriculture type	ERW lifecycle	Sex	Age	Participants with a connection to agriculture
Llandrindod Wells, mid-Wales (LW)	Upland grazing	Near to demonstrator site and quarry. Near to watercourse.	F = 5 M = 5	18–25 = 6 26–35 = 0 36–45 = 1 46–55 = 0 56–65 = 1 66–75 = 1 75+ = 1	*N* = 3
Okehampton, Devon, Southwest England (OK)	Pasture	Near to demonstrator site and quarry. Near to watercourse.	F = 5 M = 4	18–25 = 1 26–35 = 1 36–45 = 2 46–55 = 0 56–65 = 4 66–75 = 1 75+ = 1	*N* = 3
Harpenden, Hertfordshire, Southeast England (HA)	Arable	Near to demonstrator site. Near to watercourse.	F = 5 M = 4	18–25 = 1 26–35 = 3 36–45 = 1 46–55 = 3 56–65 = 0 66–75 = 1 75+ = 0	*N* = 0
Beverley, Yorkshire, Northeast England (BV)	Arable	Near to multiple watercourses. Near to coast. Near to dockland. Potential early ERW deployment.	F = 5 M = 5	18–25 = 2 26–35 = 0 36–45 = 1 46–55 = 2 56–65 = 4 66–75 = 1 75+ = 0	*N* = 2
Shrewsbury, Shropshire, East Midlands, England (SH)	Arable and Pasture	Near to quarry. Near to watercourse. Potential early ERW deployment.	F = 5 M = 5	18–25 = 2 26–35 = 3 36–45 = 1 46–55 = 1 56–65 = 1 66–75 = 1 75+ = 1	*N* = 0

## Data Availability

The data are not publicly available as they contain information that could compromise research participants’ privacy and consents.
